# Integration Analysis of Hair Follicle Transcriptome and Proteome Reveals the Mechanisms Regulating Wool Fiber Diameter in Angora Rabbits

**DOI:** 10.3390/ijms25063260

**Published:** 2024-03-13

**Authors:** Dongwei Huang, Haisheng Ding, Yuanlang Wang, Xiaofei Wang, Huiling Zhao

**Affiliations:** Anhui Provincial Key Laboratory of Livestock and Poultry Product Safety Engineering, Institute of Animal Husbandry and Veterinary Medicine, Anhui Academy of Agricultural Sciences, Hefei 230031, China; hdwscience@163.com (D.H.); dinghs123@163.com (H.D.); wangyuanlang@126.com (Y.W.); hdwwxf@163.com (X.W.)

**Keywords:** fiber diameter, hair follicles, integration analysis, proteomic, Angora rabbits

## Abstract

Fiber diameter is an important characteristic that determines the quality and economic value of rabbit wool. This study aimed to investigate the genetic determinants of wool fiber diameter through an integration analysis using transcriptomic and proteomic datasets from hair follicles of coarse and fine wool from Angora rabbits. Using a 4D label-free technique, we identified 423 differentially expressed proteins (DEPs) in hair follicles of coarse and fine wool in Angora rabbits. Eighteen DEPs were examined using parallel reaction monitoring, which verified the reliability of our proteomic data. Functional enrichment analysis revealed that a set of biological processes and signaling pathways related to wool growth and hair diameter were strongly enriched by DEPs with fold changes greater than two, such as keratinocyte differentiation, skin development, epidermal and epithelial cell differentiation, epidermis and epithelium development, keratinization, and estrogen signaling pathway. Association analysis and protein–protein interaction network analysis further showed that the keratin (KRT) family members, including KRT77, KRT82, KRT72, KRT32, and KRT10, as well as CASP14 and CDSN, might be key factors contributing to differences in fiber diameter. Our results identified DEPs in hair follicles of coarse and fine wool and promoted understanding of the molecular mechanisms underlying wool fiber diameter variation among Angora rabbits.

## 1. Introduction

Animal fibers are extensively utilized in, and serve as an important agricultural commodity for, global textile industries [1,2,3]. Rabbit fibers, being one of the most favored natural fibers worldwide, possess significant economic value within the textile industry. The annual wool yield of rabbit wool ranks third among animal fibers globally, following sheep wool and mohair [4]. Fiber diameter stands out as a primary economic trait determining the quality of rabbit wool and influencing profit returns for wool producers [5,6,7]. Given its high heritability (0.59), fiber diameter is greatly influenced by genetic factors [8]. Over the past two decades, with an increase in the demand for fine wool from Angora rabbits, the cultivation of rabbits with fine wool has become a priority in Angora rabbit breeding. Therefore, comprehending the genetic mechanisms underlying fiber diameter in Angora rabbits holds paramount importance in enhancing the economic value of rabbit wool and facilitating advancements in fine-wool Angora rabbit breeding.

Wool fibers are derived from hair follicles, which are embedded in the skin of animals, through intricate biological processes [9,10]. Rabbit fibers can be categorized into three types: guard, awn, and down hairs [4]. Guard hairs are coarse fibers with a diameter of 50–60 μm and originate from the central primary hair follicles. Down hairs are fine fibers with a diameter of 15 μm and arise from secondary hair follicles [4,9,11,12]. The diameters of coarse fibers and primary hair follicles significantly exceed those of fine fibers and secondary hair follicles [13]. Fiber diameter is not determined by embryonic hair follicle precursors, but rather by specific genes expressed during the formation of the follicle [14]. In our previous study, we analyzed the fiber diameter of coarse and fine wool as well as the morphology and transcriptome of hair follicles from coarse and fine fibers in Angora rabbits [13]. The morphology of the secondary hair follicle plays a crucial role in determining wool quality [15]. Wool growth is regulated by various molecules and signaling pathways, including fibroblast growth factor (FGF), epidermal growth factor (EGF), insulin-like growth factor 1 (IGF1), as well as Sonic Hedgehog (Shh) and MAPK signaling [16,17,18]. Polymorphisms in *FGF5*, slit guidance ligand 3 (*SLIT3*), and zinc finger protein 280B (*ZNF280B*) have been associated with variations in wool fiber diameter [19,20,21]. Proteins constitute the fundamental components of hair fibers, with wool primarily consisting of proteins that account for 90–95% of the fiber [22,23]. Wool comprises two major types of proteins: intermediate filament keratins (KRTs) and keratin-associated proteins (KAPs) [24]. The KRT and KAP families comprise major structural proteins that contribute to cuticle structure and fiber characteristics, thereby determining the quality of the fiber structure and characteristics of general hair in mammals, as well as enabling a greater understanding of these properties [1,25]. Wool growth involves both spatially and temporally dependent aggregation and expression of keratins [26]. Previous studies have revealed the contribution of lipids and carbohydrates to the composition and structure of wool [25,27], which are also present in dissected hair follicles [28].

In recent years, proteomics has been widely used in all areas of animal sciences, including the investigation of wool protein composition and its relationship with wool quality [2,29,30,31]. For instance, the characterization of skin protein profiles has been employed to explore the genetic mechanisms underlying wool production and traits in sheep of different sexes [32,33]. Through an integrated analysis of transcriptomic and proteomic data, Liu et al. [34] revealed the potential significance of the collagen alpha family proteins in goat hair follicle development and wool bending. Zhao et al. [35] systematically investigated the genetic determinants influencing fiber diameter in Tibetan cashmere goats using integrated proteomic and transcriptomic datasets. While numerous studies have focused on sheep and goats, limited information is available regarding hair follicle proteomes in Angora rabbits with coarse or fine wool. Therefore, this study aims to conduct a comparative analysis of hair follicle proteomes between coarse and fine wool of Angora rabbits using a 4D label-free quantitative approach along with an integrative analysis of transcriptome and proteome data.

## 2. Results

### 2.1. Identification of Proteins in Hair Follicles of Coarse and Fine Wool

To gain a comprehensive understanding of the proteome profiles potentially associated with differences in fiber fineness, we employed a label-free proteomic approach to investigate proteomes of hair follicles from coarse and fine wools of Angora rabbits. The results revealed that out of 1,167,434 secondary spectra, 83,708 spectra were successfully matched. A total of 2275 proteins were identified, with quantification performed for 1431 proteins (Table 1). The molecular weights of these proteins were mainly in the 10- to 70-kDa range (Figure S1A). In addition, the distribution of the number of peptides in proteins showed that most proteins contained more than two specific peptides (Figure S1B). Consequently, the identification of proteins demonstrated the accuracy and reliability of both the quantitative and qualitative results and provided the basis for the selection of differential proteins.

### 2.2. Identification of Differential Proteins in Hair Follicles of Coarse and Fine Wool

**Figure 1:**
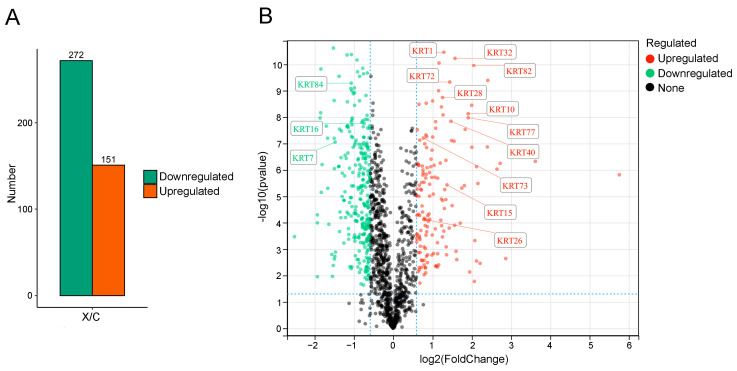
Analysis of differentially expressed proteins in the hair follicles of coarse and fine wool from rabbits. (**A**) Number of upregulated and downregulated proteins. Orange, increased expression; green, decreased expression. (**B**) Volcano plot of differentially expressed proteins. C, coarse wool group; X, fine wool group; red, increased expression; green, decreased expression; black, none.

To explore protein differences in the hair follicles between coarse and fine wool, we compared the DEPs between the two groups. DEPs were identified using fold change > 1.5 or <0.67 and *p* < 0.05 as screening criteria, resulting in the identification of 423 significant DEPs in hair follicles of coarse and fine wool samples. In comparison to the coarse (control) group, 151 proteins were upregulated and 272 proteins were downregulated in the fine (experimental) group (Figure 1A). Among the 423 DEPs, the KRT protein family, including KRT82, KRT77, KRT72, KRT32, KRT73, KRT84, KRT7, KRT26, KRT10, KRT16, KRT15, KRT28, KRT40, and KRT1, were identified (Table 2). Of these, eleven and three proteins were upregulated and downregulated in the fine wool group, respectively. The volcano plot in Figure 1 reveals the distribution of, and changes in, all DEPs from the coarse and fine wool groups, where these 14 KRT proteins were labeled (Figure 1B). These results demonstrate that the KRT protein family is a potentially important regulator that determines the structure of fine fibers in rabbits.

### 2.3. Functional Enrichment Analysis of DEPs from Hair Follicles of Coarse and Fine Wool

**Figure 2:**
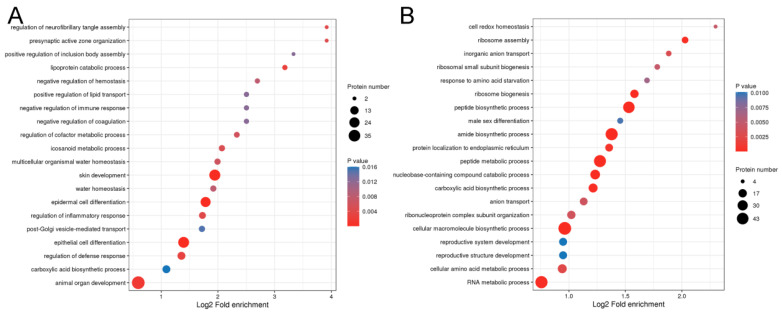
Gene ontology analysis of differentially expressed proteins in the hair follicles of coarse and fine wool from rabbits. (**A**) Upregulated proteins. (**B**) Downregulated proteins. *p* < 0.05 was considered significantly different.

**Figure 3:**
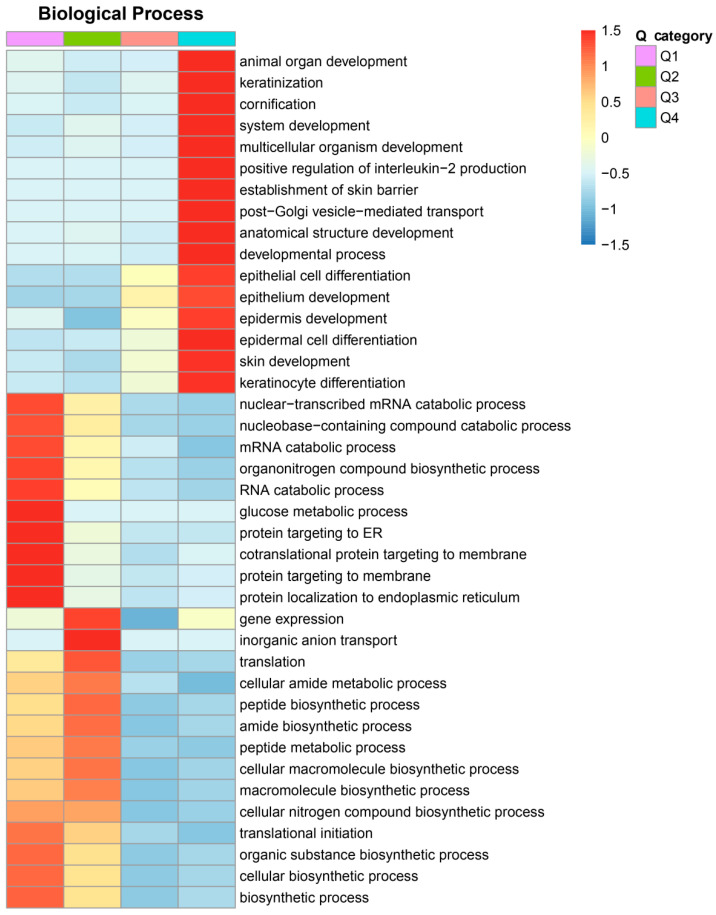
Heatmap analysis of the differentially expressed proteins with different fold changes. Relevant functions in different Q groups were gathered together. Red, strong enrichment; blue, weak enrichment. Q1 < 0.5, 0.5 < Q2 < 0.667, 1.5 < Q3 < 2.0, and Q4 > 2.0.

To investigate the potential function of DEPs in fiber fineness in rabbits, we conducted functional enrichment analysis on the genes encoding DEPs (Figure 2). Gene ontology (GO) analysis discriminated between upregulated and downregulated proteins. As shown in Figure 2A, the upregulated proteins were significantly enriched in epithelial cell differentiation (29 DEPs), epidermal cell differentiation (23 DEPs), skin development (30 DEPs), positive regulation of lipid transport (3 DEPs), and lipoprotein catabolic process (3 DEPs). However, the downregulated proteins were mainly involved in common structural or functional processes (Figure 2B). We further divided the DEPs into four groups (Q1, Q2, Q3, and Q4) according to fold change, as shown in Figure S2. GO classification enrichment was then performed for each group to explore correlations in the function of proteins with different fold changes. GO terms related to biological processes were enriched by DEPs with different fold changes (Figure 3). Proteins with fold changes > 2 were strongly enriched in keratinocyte differentiation, skin development, epidermal cell differentiation, epidermal development, epithelium development, epithelial cell differentiation, and keratinization. However, the other DEPs, with a fold change < 2, were weakly enriched in biological processes related to hair fibers.

**Figure 4:**
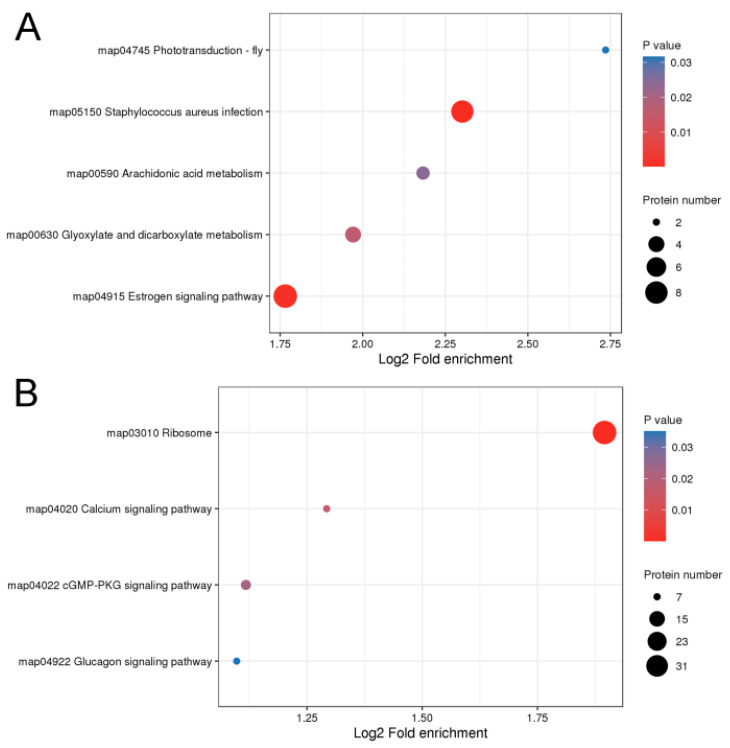
Enriched Kyoto Encyclopedia of Genes and Genomes pathways of the differentially expressed proteins in the hair follicles of coarse and fine wool. (**A**) Upregulated proteins. (**B**) Downregulated proteins. *p* < 0.05 was considered significantly different.

Kyoto Encyclopedia of Genes and Genomes (KEGG) enrichment analysis was conducted to further characterize the biological functions of the identified DEPs (Figure 4). The upregulated proteins were involved in the phototransduction-fly (CALML5 and ACTB) and estrogen signaling pathways (KRT26, KRT10, CALML5, KRT32, KRT15, KRT28, and KRT40), and the downregulated proteins were involved in the ribosome, calcium, and cGMP-dependent protein kinase or protein kinase G (cGMP-PKG) signaling pathways. In particular, the estrogen signaling pathway was strongly enriched by upregulated proteins (KRT10, KRT32, KRT15, KRT28, and KRT40) with a fold change > 2 (Figure S3). These findings showed that the upregulated proteins, especially proteins with a fold change > 2, were important in determining fiber diameter in rabbits.

### 2.4. Integrated Analysis of Transcriptome and Proteome of Hair Follicles from Coarse and Fine Wool

**Figure 5:**
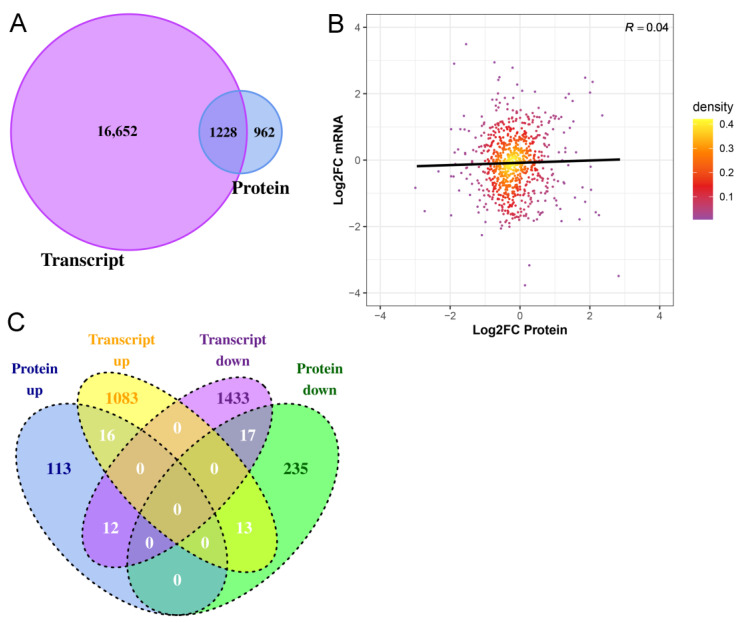
Comparative analysis of the transcriptome and proteome of the hair follicles from coarse and fine wool. (**A**) Quantitative comparison of transcriptome and proteome. (**B**) Comparison of the expression between transcriptome and proteome. The x-axis and y-axis indicate the Log2 Fold Change (Log2FC) of the proteomic and transcriptomic profile, respectively. (**C**) Venn plot of up-regulated and down-regulated DEPs and differentially expressed genes (DEGs) detected at both the protein and transcript levels in comparison groups. The transcriptome data used in this analysis was taken from our previous study [13].

**Figure 6:**
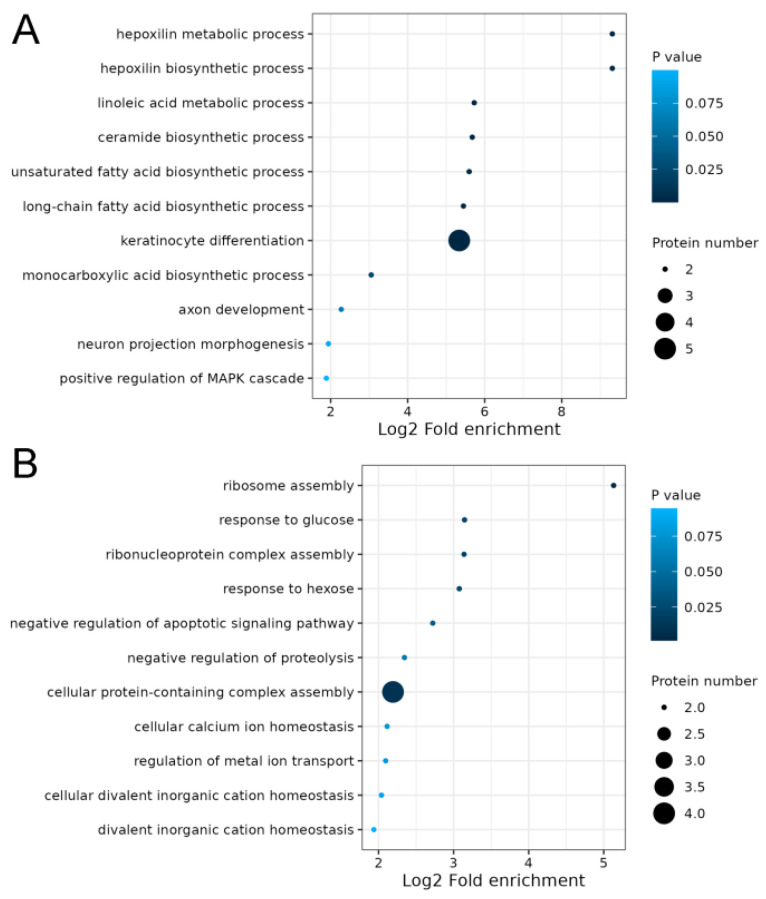
Gene ontology analysis of proteins differentially expressed at the transcriptomic and proteomic levels. (**A**) Upregulated proteins. (**B**) Downregulated proteins.

The transcriptome data of the hair follicles of coarse and fine wool from our previous study were used for integrated analyses [13]. A total of 1228 genes were quantified at both the transcriptomic and proteomic levels (Figure 5A). Subsequently, the correlation between gene expression and protein expression was examined (Figure 5B). The Pearson correlation coefficient was found to be very low (0.04), indicating that mRNA and protein expression levels in Angora rabbit hair follicles were not always positively correlated. Furthermore, the changing trends of all DEPs at both the transcriptomic and proteomic levels were analyzed. Based on mRNA and protein expression levels, a total of 33 proteins (Table S1), including 16 upregulated and 17 downregulated proteins, exhibited concordant trends at both the transcriptomic and proteomic levels (Figure 5C). Thirteen DEPs showed increased expression at the transcriptomic level but decreased expression at the proteomic level, while twelve DEPs displayed downregulation at the transcriptomic level but upregulation at the proteomic level. Notably, KRT72, KRT73, KRT77, KRT82, and KRT32 were upregulated at both the transcriptomic and proteomic levels. GO analysis demonstrated that keratinocyte differentiation (KRT73, KRT77, KRT82, KRT72, and KRT32), unsaturated fatty acid biosynthetic processes (ALOX12B and ALOXE3), and long-chain fatty acid biosynthetic processes (ALOX12B and ALOXE3) were significantly enriched in proteins upregulated at both the transcriptomic and proteomic levels (Figure 6A, Table S2). Proteins that were downregulated at the transcriptomic and proteomic levels were not involved in regulating hair follicle development (Figure 6B, Table S3).

### 2.5. Protein-Protein Interaction (PPI) Networks

**Figure 7:**
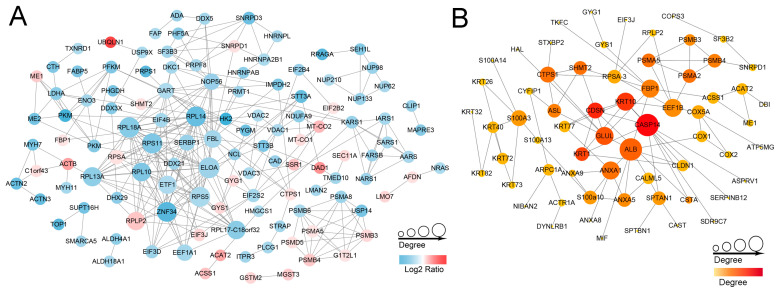
Protein–protein interaction networks of differentially expressed proteins in coarse (control) and fine (experimental) wool groups. (**A**) Differentially expressed proteins. Red, upregulated proteins; blue, downregulated proteins. A larger circle indicates a stronger interaction with more genes; a darker color indicates a larger fold change. (**B**) Upregulated proteins. Larger circle sizes and darker colors indicate stronger interactions with more genes.

We further investigated the mechanism of DEPs involved in regulating the fineness of hair fibers and revealed functional interactions among the DEPs. PPI network analysis of the DEPs in the STRING database was performed. Sequential visualization was performed using Cytoscape software (version 3.8.2). In total, 120 proteins formed a tightly connected network with 284 edges (Figure 7A). Among these 120 DEPs, 30 were upregulated, while 90 were downregulated. The hub nodes, including ELOA, RPL18A, RPS11, RPS5, RPL14, ZNF34, RPL13A, and RPL10, exhibited more than 40 connections and all belonged to ribosomal proteins that showed downregulation in the ribosome pathway. Additionally, we analyzed the upregulated proteins and the relationships between them are shown in Figure 7B. Hub proteins such as CASP14, GLUL, KRT10, CDSN, and ALB, all with high degree values, were identified.

### 2.6. Validation of Proteins via Parallel Reaction Monitoring (PRM)

**Figure 8:**
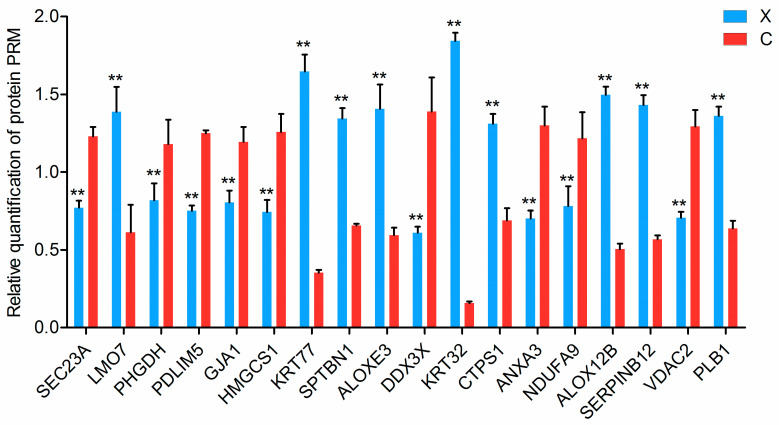
Verification of the differentially expressed proteins using PRM. X, hair follicle of the fine wool group; C, hair follicle of the coarse wool group. ** *p* < 0.01.

According to the integrated analysis results, we selected and assessed the 33 proteins that exhibited consistent differential expression patterns at both the transcriptomic and proteomic levels in coarse and fine wool samples. Nineteen proteins were further validated using PRM-targeted proteomics. The results showed that 18 proteins were successfully quantified (Figure 8, Table S4). The relative abundance level of all of the 18 proteins (SEC23A, LMO7, PHGDH, PDLIM5, GJA1, HMGCS1, KRT77, SPTBN1, ALOXE3, DDX3X, KRT32, CTPS1, ANXA3, NDUFA9, ALOX12B, SERPINB12, VDAC2, and PLB1) showed similar upregulation or downregulation trends in both 4D label-free-based proteomics and PRM, confirming the reliability of our proteomics data. 

## 3. Discussion

Rabbit hair is characterized by its antistatic properties, softness, glossiness, and durability, and as a result, it is usually chosen to produce luxury textile materials [4]. It is also the core of the third-largest animal fiber industry worldwide [36]. Fiber diameter has important commercial value in fiber production and greatly influences the quality of rabbit hair. In addition, fiber diameter determines the processing performance and end use of wool, as well as its market price [37,38]. 

In our study, we performed a proteome-wide analysis of hair follicles from coarse and fine wool of Angora rabbits and identified a set of functional proteins involved in regulating the diameter of the wool fibers. The present study showed that 423 proteins (151 upregulated and 272 downregulated) were differentially expressed in hair follicles of coarse and fine wool. The GO enrichment analysis revealed that many upregulated proteins were involved in epithelial and epidermal cell differentiation, skin development, positive regulation of lipid transport, and lipoprotein catabolism. In particular, the upregulated proteins with fold changes > 2 exhibited significant enrichment in biological processes related to hair follicle development, such as keratinocyte differentiation, skin development, epidermal cell differentiation, epidermal and epithelial development, epithelial cell differentiation, and keratinization. Conversely, only a limited number of downregulated proteins in the fine wool group participated in the above biological processes to regulate fiber structure. Consequently, we speculate that the upregulated proteins in the fine wool group, particularly those displaying fold changes > 2, played pivotal roles in determining the structure of fine fibers in Angora rabbits. Skin development and epidermal and epithelial cell differentiation were significantly enriched by KRTAP24-1, KRT82, KRT77, KRT72, KRT10, KRT32, KRT15, KRT28, and KRT40, all with fold changes > 2. KRT and KAP proteins are the main structural proteins of wool, forming a matrix between the keratin intermediate filaments in the fiber [39,40]; they also play a role in determining hair fiber quality in mammals [41]. It has been reported that the caprine *KRTAP24-1* gene serves as a marker for fine cashmere fiber by influencing its diameter [6]. KRT10 is expressed early during the differentiation of keratinocytes and is essential for epidermal integrity [42]. KRT77, a member of the type II keratin family, plays an important role in epidermal and coat formation [43]. Furthermore, differential abundance of KRT40 has been observed between cashmere and guard fiber in sheep and goats [1]. Collectively, these findings highlight the significance of these differentially expressed KRT proteins as important structural components that may contribute to variations in hair diameter among Angora rabbits. 

The KEGG-enriched upregulated proteins mainly fall into the phototransduction-fly and estrogen signaling pathways, while the downregulated proteins are mainly involved in the ribosome, calcium, and cGMP-PKG signaling pathways. The phototransduction-fly signaling pathway is a photoperiodic pathway involved in regulating biological processes [44]. Red light can promote the growth of rabbit hair and the development of secondary hair follicles, increase the percentage of fine hair, and reduce the fineness of fine hair; green light improves the fineness of coarse hair and hinders hair follicle development in rabbits [45]. It is worth noting that the phototransduction-fly pathway is enriched by upregulated proteins (CALML5 and ACTB), with the *CALML5* gene being an essential epidermal regulator required for late epidermal differentiation [46]. Therefore, it can be inferred that the phototransduction-fly pathway plays a crucial role in regulating rabbit hair quality and hair growth. Estrogen is an important modulator of skin physiology and hair growth [47,48]. Proteins with fold changes > 2 (KRT10, KRT32, KRT15, KRT28, and KRT40) were strongly enriched in the estrogen signaling pathway. These proteins are important structural proteins in wool. This finding suggests that the estrogen signaling pathway is involved in regulating the structural components of fine wool in Angora rabbits. Both our present study and previous research have shown substantial enrichment of DEPs and DEGs related to ribosome pathways between coarse and fine wool hair follicles [13]. Similarly, ribosome pathways were also significantly enriched among DEGs from two groups of Gansu alpine Merino sheep with different wool fiber diameters [49]. A defect in ribosomes has been shown to alter cell fate from a hair cell to a non-hair cell [50,51]. Therefore, we hypothesize that the phototransduction-fly signaling pathway, along with estrogen and ribosome pathways, potentially play vital roles in controlling hair fineness in Angora rabbits. Calcium and cGMP-PKG are key signal transduction pathways involved not only in regulating Ca^2+^ concentration but also in maintaining ion homeostasis by balancing potassium, sodium, and anions in organisms [52,53,54,55]. This indicates that a stronger regulation of ion homeostasis occurs in the hair follicles of coarse wool. 

Association analysis of the transcriptome and proteome is an efficient strategy for understanding the principles of gene expression control and can provide more comprehensive gene expression information [56]. Correlation analysis revealed that 1228 genes were expressed at both the transcriptomic and proteomic levels in hair follicles between coarse and fine wool. However, our study demonstrated a low correlation (0.04) between transcriptomic and proteomic profiles. Among these, only 33 differentially expressed proteins exhibited consistent trends at both the transcriptional and translational levels. Previous studies have also reported a limited correlation between the transcriptome and proteome [57,58]. This discrepancy may arise from the fact that changes in mRNA abundance do not necessarily translate directly into corresponding alterations in protein levels. Genetic variability that does not alter transcription levels may alter protein functions and thus potentially alter phenotypes [59]. Moreover, it is important to consider that individual genes typically generate diverse transcripts and proteoforms [60,61], which can lead to variations in relative protein expression levels and potentially functional proteins, based on their respective mRNA abundance [62,63]. 

In addition, GO terms associated with hair growth were enriched in proteins upregulated at the transcriptomic and proteomic levels, such as in keratinocyte differentiation, unsaturated fatty acid biosynthetic processes, and long-chain fatty acid biosynthetic processes, which directly or indirectly regulate hair structures. For instance, keratinocytes play important roles in follicle induction and hair growth [64,65]. Unsaturated fatty acid biosynthesis is also involved in hair metabolism [66]. Biosynthesis of long-chain fatty acids is highly important for skin function as it serves as a structural component that is not bound to membranes; it contributes to the development and maintenance of both hair and skin function [67]. The upregulated proteins, including KRT73, KRT77, KRT82, KRT72, KRT32, ALOX12B, and ALOXE3, were involved in these biological processes, indicating their significant contribution to the difference in diameter between coarse and fine wool observed in Angora rabbits.

Finally, PPI analysis of the upregulated proteins showed that the hub nodes CASP14, KRT10, and CDSN were associated with wool traits and hair growth. For instance, CASP14 and KRT10 have been implicated in keratinocyte differentiation [42,68]. CDSN is expressed in the epidermis and inner root sheath of hair follicles and plays a key role in hair physiology [69]. We subsequently verified these results using PRM-targeted proteomics and further demonstrated the reliability of our data.

## 4. Materials and Methods

### 4.1. Animals and Sample Collection

Twelve healthy 1-year-old female Angora rabbits from the Animal Husbandry Institute of the Anhui Academy of Agriculture Sciences were used in this study. The Angora rabbits were divided into four groups. Each group contained three rabbits from the same parent that were raised under the same conditions (ambient temperature and natural photoperiod) and fed based on appetite, with drinking water supplied using an automatic water feeder. The coarse and fine wool fibers of each Angora rabbit were pulled from the roots from the back of the body in the spring, when wool fiber grew to 73 days after shaving. The hair follicles of coarse and fine wool were cut and placed in a 2.0 mL freezing tube, snap-frozen in liquid nitrogen, and then stored at −80 °C for subsequent experiments.

### 4.2. Hair Follicle Protein Extraction and Tryptic Digestion

The hair follicle samples of coarse and fine wool fibers were ground into powder with liquid nitrogen and then transferred to centrifuge tubes. Four volumes of lysis buffer (8 M urea and 1% protease inhibitor cocktail) were added to the powder, followed by three treatments with a high-intensity ultrasonic processor (Scientz). The remaining debris was removed by centrifugation at 12,000× *g* at 4 °C for 10 min. The supernatant was collected, and the protein concentration was determined using a Bicinchoninic Acid Assay (BCA) kit and then normalized to 1 mg/mL. Then, protein samples from each group were mixed into one biological replicate. Finally, four biological replicates were obtained from the coarse and fine wool groups, respectively. 

The protein solution was reduced using 5 mM dithiothreitol for 30 min at 56 °C and alkylated with 11 mM iodoacetamide for 15 min at room temperature in darkness. The protein sample was diluted with 100 mM triethylammonium bicarbonate (TEAB) to a urea concentration of <2 M. Trypsin solution was then added at a trypsin-to-protein mass ratio of 1:50 for the first digestion overnight, and at a ratio of 1:100 for the second 4 h digestion. 

### 4.3. Liquid Chromatography Tandem Mass Spectrometry Analysis

The tryptic peptides were dissolved in solvent A (0.1% formic acid, 2% acetonitrile/water), directly loaded onto a home-made reverse-phase analytical column (length, 25 cm; diameter, 75/100 μm). The gradient consisted of increasing solvent B (0.1% formic acid, 90% acetonitrile) from 6% to 24% over 70 min, from 24% to 35% over 14 min, then to 80% over 3 min, remaining at 80% for a further 3 min, at a constant flow rate of 450 nL/min on a nanoElute UHPLC system (Bruker Daltonics, Billerica, MA, USA). The separated peptides were injected into the capillary ion source for ionization and then into the timsTOF Pro mass spectrometer (Bruker Daltonics, Billerica, MA, USA) for analysis. The ion source voltage was set at 1.60 kV and the peptide parent ions and their secondary fragments were detected and analyzed using a high-resolution TOF. The mass spectrometry scan range was set to 100–1700 *m*/*z*. Data acquisition was conducted using the parallel accumulation serial fragmentation (PASEF) mode. The 10-fold PASEF mode acquisition of mother ions was performed after first-level mass spectrum acquisition, using a cycle window time of 1.17 s. For secondary spectra with charge numbers of zero to five, the dynamic exclusion was set at 30 s.

### 4.4. Data and Bioinformatics Analysis

The resulting liquid chromatography-tandem mass spectrometry (LC-MS/MS) data were processed using the MaxQuant search engine version 1.6.15.0. Search parameter settings were as follows: database for Oryctolagus_cuniculus_9986_PR_20220422 (41,459 entries). Trypsin/P is designated as an enzyme that allows up to two deletions. The mass tolerance for the precursor ions was set to 20 ppm in the first search and to 5 ppm in the main search. The mass tolerance for the fragment ions was set to 0.02 Da. Carbamidomethylation on Cys was specified as a fixed modification, and acetylation at the protein N-terminus and oxidation on Met were specified as variable modifications. The false discovery rate (FDR) was adjusted to <1%, and the minimum score of modified peptides was set to >40. Proteins with a fold change > 1.5 or < 0.67 and statistical significance (*p* < 0.05) were considered DEPs. 

The protein functions were analyzed based on searches against the gene ontology (GO; https://geneontology.org/, accessed on 21 December 2021) and Kyoto Encyclopedia of Genes and Genomes (KEGG; www.kegg.jp/feedback/copyright.html, accessed on 17 September 2021) databases [70]. A *p* value < 0.05 was considered significantly different for GO terms and pathways. A protein-protein interaction (PPI) network was constructed with a confidence score ≥ 0.7 using STRING version 11.0 [71] and visualized and analyzed using Cytoscape software version 3.8.2 (http://www.cytoscape.org/, accessed on 15 July 2023). The hub genes were identified using the plug-in cytoHubba in Cytoscape software. Correlation analysis of genes and proteins was performed using the R function core test. The transcriptome data from the hair follicles of coarse and fine wool were from our previous study [13].

### 4.5. Validation of Protein Expression Levels Using Parallel Reaction Monitoring

Protein extraction and digestion were performed as described above. Proteins (200 μg) from each sample [33] were prepared for parallel reaction monitoring (PRM) detection. The mobile phase was run at a constant flow rate of 1000 L/min in gradient mode. Mobile phase A contained 0.1% formic acid and 2% acetonitrile, while mobile phase B contained 0.1% formic acid and 90% acetonitrile. The liquid phase gradient setting was as follows: from 0 to 40 min, the gradient was 6% to 20% B; from 40 to 52 min, the gradient was 20% to 30% B; from 52 to 56 min, the gradient was 30% to 80% B; and from 56 to 60 min, the gradient was 80% B. The flow rate was maintained at 450 nL/min. After isolation, the peptides were subjected to an NSI source followed by tandem mass spectrometry (MS/MS) in Q Exactive HF-X (Thermo, Grand Island, NY, USA). The electrospray voltage applied was 2.1 kV. A primary mass spectrometry scan range was set at 440–1265 *m*/*z*, with a resolution of 30,000. Automatic gain control (AGC) was set at 3 × 10^6^, and the maximum ion implantation time (MIT) was 50 ms. For the secondary mass spectrometry Orbitrap scan, the resolution was set at 17,500, AGC was set at 2 × 10^5^, and MIT was set at 220 ms. The isolation window for MS/MS was set at 1.4 *m*/*z*. 

The resulting MS data were processed using Skyline version 3.6 software. For the peptide settings, the enzyme was set as trypsin [KR/P], and the maximum missed cleavage was set as two. The peptide length was set as seven to twenty-five, and fixed modification was set as the carbamidomethyl on Cys. The transition settings were as follows: precursor charges were set as two and three, ion charges were set as one, and ion types were set as b and y. The product ions were set from ion three to the last ion, and the ion-match tolerance was set as 0.02 Da. The FDR for protein identification was set at 1%.

### 4.6. Statistical Analysis

The data were expressed as means ± standard deviation and determined using GraphPad Prism 5 (GraphPad Software, Inc., La Jolla, CA, USA) in this study. A student’s *t*-test was employed to analyze differences between two groups. The threshold of *p* < 0.05 was considered statistically significant.

## 5. Conclusions

In summary, we characterized the differences in protein abundance between hair follicles of coarse and fine wool from Angora rabbits. Through integrated analysis of transcriptome and proteome, we identified functional and regulatory networks. Our findings revealed proteins, such as KRTAP24-1, KRT10, KRT32, KRT15, KRT28, KRT40, KRT73, KRT77, KRT82, KRT72, CASP14, and CDSN, which may be associated with wool fiber diameter. This study provides valuable transcriptomic and proteomic expression datasets that contribute to our understanding of the regulatory mechanisms underlying the differences in fiber diameters between coarse and fine wool in Angora rabbits. Further studies are warranted to elucidate the regulatory mechanisms of candidate functional proteins on wool fiber diameter. 

## Figures and Tables

**Table 1:** Summary of protein identification.

Total Spectra	Matched Spectra	Peptides	Unique Peptides	Identified Proteins	Quantifiable Proteins
1,167,434	83,708	14,655	12,463	2275	1431

**Table 2:** Expression levels of differentially expressed KRT proteins between coarse and fine wool of Angora rabbits.

Protein Accession	Gene Name	X Average	C Average	X/C Ratio	X/C *p* Value	Regulation Type
G1SDF4	KRT82	1.6102455	0.3897545	4.131	1.10187 × 10^−10^	Up
G1SHX4	KRT84	0.646035	1.35396525	0.477	5.18384 × 10^−10^	Down
G1SHZ4	KRT7	0.5288665	1.47113325	0.359	8.82596 × 10^−8^	Down
G1SKE3	KRT77	1.579011	0.420989	3.751	1.05656 × 10^−8^	Up
G1SUH1	KRT72	1.4606215	0.5393785	2.708	4.71087 × 10^−10^	Up
G1SWB4	KRT26	1.2793635	0.7206365	1.775	7.79235 × 10^−5^	Up
G1T1V0	KRT10	1.57839425	0.42160575	3.744	7.48266 × 10^−9^	Up
G1T4P0	KRT32	1.49585425	0.50414575	2.967	5.87822 × 10^−11^	Up
G1T4R6	KRT16	0.74950175	1.25049825	0.599	1.71792 × 10^−8^	Down
G1T6X1	KRT15	1.44077525	0.559225	2.576	3.67408 × 10^−6^	Up
G1T8T1	KRT28	1.40851325	0.5914865	2.381	1.81837 × 10^−9^	Up
G1T9T2	KRT40	1.46848275	0.53151725	2.763	1.43752 × 10^−8^	Up
G1TDN8	KRT73	1.25889925	0.74110075	1.699	6.19365 × 10^−8^	Up
G1U9I8	KRT1	1.4189405	0.5810595	2.442	3.43761 × 10^−11^	Up

## Data Availability

The data are available from the corresponding authors upon reasonable request.

## Supplementary Materials

The following supporting information can be downloaded at https://www.mdpi.com/article/10.3390/ijms25063260/s1.
