# Supplementary material for: Integration Analysis of Hair Follicle Transcriptome and Proteome Reveals the Mechanisms Regulating Wool Fiber Diameter in Angora Rabbits
Source: Int J Mol Sci. 2024 Mar 13;25(6):3260. doi: 10.3390/ijms25063260 (PMC10970426; doi:10.3390/ijms25063260)
Supplement: Supplementary file 1 [file ijms-25-03260-s001.zip › Supplementary Figures.pdf]

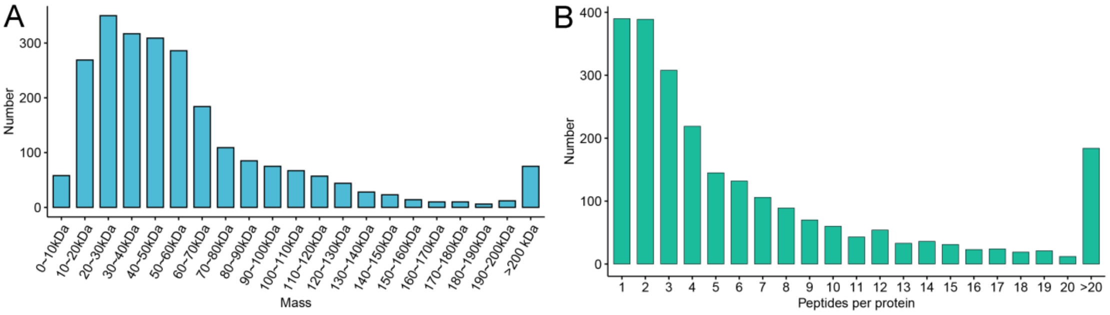

Figure S1. Identification of proteins in hair follicles of coarse and fine wool. (**A**) Molecular weight distribution of protein. Abscissa, molecular weight of the identified proteins; ordinate, number of the identified proteins. (**B**) Distribution of peptides in proteins.

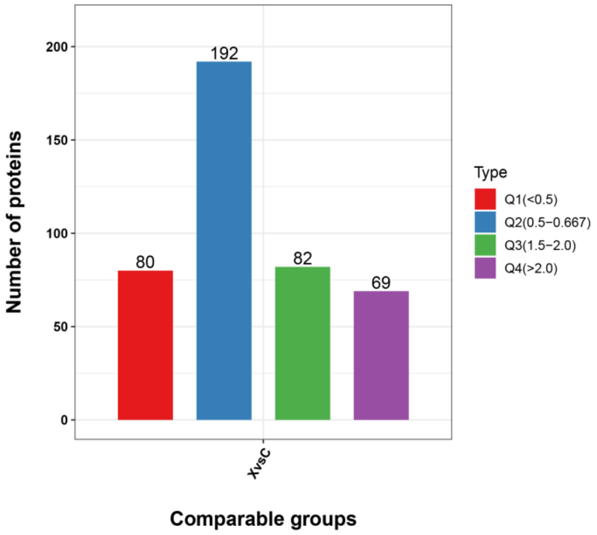

Figure S2. Proteins grouped according to the values of fold change.

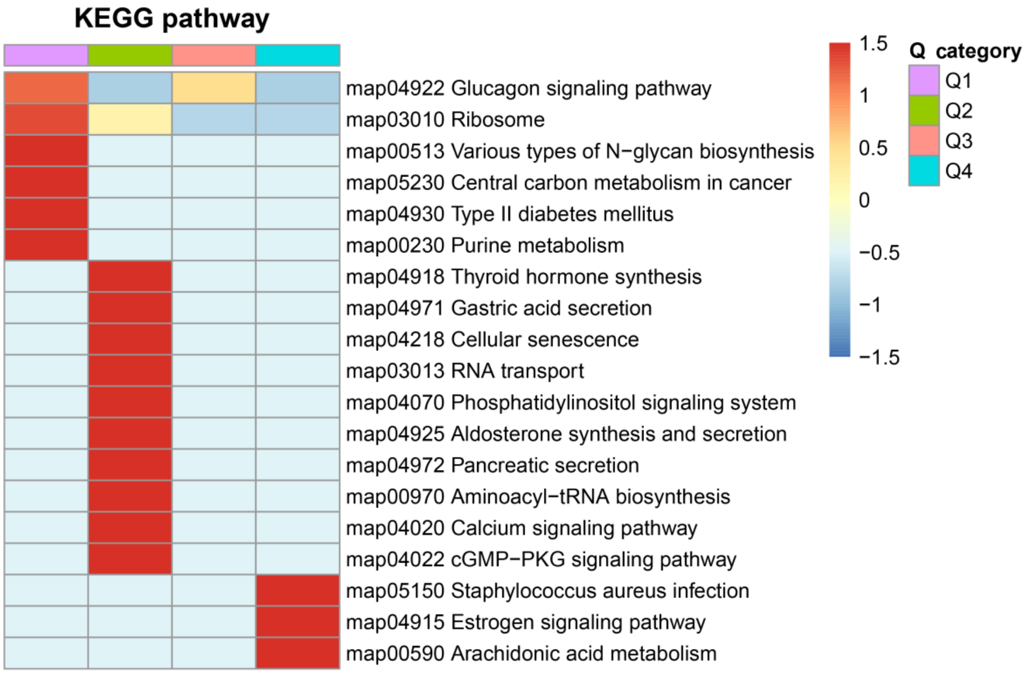

Figure S3. Heatmap analysis of differentially expressed proteins with different fold changes, and relevant Kyoto Encyclopedia of Genes and Genomes pathways in different Q groups are gathered together. Red, strong enrichment; blue, weak enrichment. Q1<0.5, 0.5<Q2<0.667, 1.5<Q3<2.0, and Q4>2.0.
